# The "Ad Hoc" Principle in Local Government

**Published:** 1907-12-21

**Authors:** 


### THE "AD HOC" PRINCIPLE IN LOCAL GOVERNMENT.

Nothing probably has given rise to greater misconception as to the province of popular control in local government than the initiation of *ad hoc* authorities.

That such authorities should struggle for their own perpetuation is not to be wondered at, and it was only to be expected that the Poor-law Unions Conference recently held should endeavour to withstand the trend of public opinion by affirming the desirability of maintaining the *status quo* in respect of the organisation of Poor-law authorities. We learn that "the suggestion that there should be one Poor-law authority for the Metropolis, and that in the provinces the County Councils should be entrusted with the work, had been considered by the Executive Council, who reported that they were of opinion that very great disadvantages would result from the transfer of the control of the Poor-law to either the County or Municipal Councils, and that they were resolved to continue to take every possible opportunity of protesting against any such change of control of Poor-law affairs. This resolution of the Council was adopted."

It will, however, require more than the complacency of the existing Poor-law authorities to persuade the public that their continuance is desirable. The disclosures of recent inquiries made by the Local Government Board into the conduct of Unions in and around the Metropolis have shaken the confidence of the public, if it ever existed, as to the fitness of Boards of Guardians for local administration. Even the most ardent democrats must recognise that nothing is better calculated to defeat the cherished end of public control than the multiplication of small local bodies having a narrow range of public interest and drawn for the most part from the less intelligent sections of the community.

Probably in this mistaken purpose more than in any other lies the cause of so much failure in local government, and especially so is it with *ad hoc* authorities. What local authorities have yet to learn is that they are elected for purposes of local administration, as Parliamentary representatives are for purposes of central government. Were this understood, the economy in local government would be immense. The larger authorities for the most part can distinguish when the supremacy of public interest lies in the acceptance of expert opinion, but the smaller bodies flounder among technical difficulties, with which it pleases them to think the amateur is better qualified to deal in virtue of some electoral legerdemain than is the expert by his training. When the areas of local government are made sufficiently large and the public interests which they control sufficiently wide, there will be some chance of public control achieving its legitimate purpose, which will be found not inconsistent with the acceptance of expert guidance. In official medical work this is especially desirable. Probably no professional territory is so ruthlessly and stupidly invaded as that of medicine, and the position of medical officers to the smaller public bodies is often pitiable. It is frequently said by officials of the larger bodies that it is their own fault if officers, be it to a Union or a health authority, fail to secure the respect of their petty masters; but this is a species of Pharisaism which arises from ignorance of the depths of incapacity and blundering petty tyranny of which small ignorant bodies are capable. Without endeavouring to anticipate its findings, it is not too much to hope that when the Royal Commission on the Poor-law reports, the last of the *ad hoc* authorities will have received its *congé*.

